# The Anisotropic Electrothermal Behavior and Deicing Performance of a Self-Healing Epoxy Composite Reinforced with Glass/Carbon Hybrid Fabrics

**DOI:** 10.3390/molecules30132794

**Published:** 2025-06-28

**Authors:** Ting Chen, Xusheng Du

**Affiliations:** School of Chemistry and Materials, Institute of Advanced Wear & Corrosion Resistant and Functional Materials, Jinan University, Guangzhou 510632, China; chenting202208@163.com

**Keywords:** composites, carbon fiber, hybrid fiber, electrical heating, deicing

## Abstract

Hybrid fiber-reinforced polymer-laminated composites are often used under icy conditions (such as for reinforcing parts in aircraft frames and bridge beams), where there is an urgent demand for deicing. In this paper, besides the different mechanical properties of laminates along the longitudinal carbon fiber (CF) and glass fiber (GF) directions, the anisotropic electrothermal behavior of a hybrid glass/carbon fiber-reinforced epoxy (GCF/EP) is also investigated, as well as its deicing performance and self-repairing capability. The surface equilibrium temperature of GCF/EP composites can conveniently be adjusted by tuning the current magnitude and its flow direction. Compared to the longitudinal CF direction of the GCF/EP, where 0.3 A was loaded to achieve a surface equilibrium temperature of 122.8 °C, a much weaker current (0.03 A) was needed to load along the longitudinal GF direction to reach almost the same temperature. However, besides the higher flexural strength and fast temperature response, along the longitudinal CF direction, the GCF/EP exhibited excellent deicing performance, including a shorter time and larger energy efficiency. Furthermore, the self-repairing ability of the GCF/EP and its effect on the deicing performance of the composite were characterized. Studying the Joule heating effect of GCF/EP composite laminates and their corresponding deicing performance lays the foundation for their design and practical application in icy environments.

## 1. Introduction

Glass/carbon fiber composites are widely used in the aerospace industry, yacht hulls, sports equipment, wind turbine blades, and the chemical engineering industries due to their excellent strength, stiffness, processability, flame retardancy, and fatigue resistance [1,2,3,4,5,6]. However, fiber composites in these fields inevitably encounter climatic extremes, including low temperatures and icy conditions [7,8]. Ice accumulation or “icing” can pose a hazard to the composite components of icebreakers, for example by damaging communication antennas, causing ladders and walkways to become slippery, and causing the malfunction of lifeboats [9]. The primary method of reducing surface icing on composite parts is deicing. Many deicing methods have been developed so far, such as techniques using deicing salt, direct hot-air heating, and electrothermal heating. In the electrothermal deicing method, an electric current is passed through an electrical heating element, melting the ice or reducing the adhesion between the ice and the surface. This method is effective and convenient to carry out, as it does not involve any physical or chemical treatment of the surface; therefore, it has great potential for practical application [10,11,12,13]. Generally, conventional electrical heating elements are made of nickel–chromium alloys and iron–chromium–aluminum alloys, and the main drawbacks of these alloys are the complexity of their fabrication process and their poor chemical resistance. Contrary to these common alloys, not only can glass fiber/carbon fiber composites be directly used as advanced engineering materials to provide mechanical support to whole structures, but they also have potential applicability in integrated electrical heating elements, harnessing their good processability, excellent chemical stability, and corrosion resistance. Unfortunately, little information on the deicing performance of practical hybrid fiber-reinforced polymer composites is currently available.

Various attempts have been made by researchers to improve the electrothermal properties and deicing performance of fiber-reinforced composites. Hong et al. [14] studied the deicing behavior of a graphene oxide-modified carbon fiber/epoxy (GO@CF/EP) composite, and the ice encased on the surface of the composite could be melted within 175 s. In a recent work, we found that a pultrusion-molded carbon fiber/epoxy composite plate (CF/EP) could reach an equilibrium temperature of 91 °C within 3 min under the application of a 5 A current [15], and that the deicing process of composites can easily be shortened by loading stronger currents. It was reported recently that about 50 mg of ice on the surface of a GO-modified CF/EP plate could be completely melted in 50 s under Joule heating at 6 V [16]. In addition, CNTs (carbon nanotubes) were also utilized as effective fillers of polymer composites to improve their electrothermal and/or deicing performance [17,18]. To afford electrical and deicing properties to a glass fiber/epoxy (GF/EP) composite, CNTs in the form of bucky paper were introduced into the composite system, thereby improving the deicing performance by shortening the heating time significantly compared to its counterparts [17]. Glass fibers are often considered a low-cost, noncombustible, inorganic material with good mechanical strength and interfacial compatibility with epoxy resins [19]. Due to the polar groups on the surface of GFs, they grant better interfacial properties to epoxy than CFs, which have superior mechanical properties along with the characteristic of chemical inertness to most polymer matrixes. To combine the advantages of these two important fiber materials, GF/CF hybrid fiber (GCF)-reinforced polymer composites have been developed to achieve the desired mechanical qualities at lower costs [1,20], which makes them more easily commercialized than their counterparts reinforced with a single type of fiber. Taking into account GFs’ and CFs’ differing mechanical and electrical characteristics, the mechanical and electrothermal properties of their composite laminates should be anisotropic, as should their deicing performance. 

More recently, self-healing fibers and their composites with various functions have attracted more and more attention. An electroluminescent fiber composite made of a nickel electrode core, a fluoropolymer interlayer, and hydrogel cladding was fabricated, and it was found that 98.6% of the initial brightness could be restored by heating it at 60 °C for 24 h after fracture [21]. A self-healing gel fiber with a good thermoelectric response was also prepared via a technique of co-axial wet-spinning, and it displayed a self-repair efficiency of 89.12% at room temperature and a high degree of sensitivity to temperature in the range of 200–400 °C, which made it suitable for the safety monitoring of firefighting clothing [22]. The gel materials in the functional fibers played an important role in their self-healing processes in these works. In one study, cellulose nanocrystal/waterborne polyurethane composites with both self-healing ability and solvent response were prepared, and the functions were realized by dynamic disulfide bonds (S-S) in the macromolecules and carbon nanofiber fillers [23]. The composites underwent a reversible color change upon immersion in solvents, which rendered them suitable for use in the fields of anti-counterfeiting and flexible photonic devices. For epoxies that are used as structural engineering materials, self-healing epoxy resins based on various covalent bonds, including dynamic disulfide bonds and/or ester bonds, have been developed [24,25,26,27,28,29,30]. These afford conventional fiber-reinforced polymer composites a self-healing ability as well. Therefore, both the mechanical and deicing recovery of healed GCF/EP are worthy of investigating. However, little research on these important and practical issues can be found in the current literature. 

In this work, the anisotropic character of the mechanical properties, electrical heating behavior, and deicing performance of GCF/EP-laminated composites will be studied. Specifically, the electrothermal behavior and cyclic heating–cooling stability of the hybrid fiber-reinforced epoxy composites across various longitudinal fiber directions will be characterized. Moreover, the deicing rate and the corresponding energy efficiency of the GCF/EP composites along both the longitudinal CF and GF directions will be measured and analyzed. Furthermore, the impact of self-healing on the restoration of both the mechanical and deicing properties of the hybrid fiber-reinforced epoxy composites will be elucidated.

## 2. Results and Discussion

### 2.1. Mechanical Property Analysis

**Figure 1:**
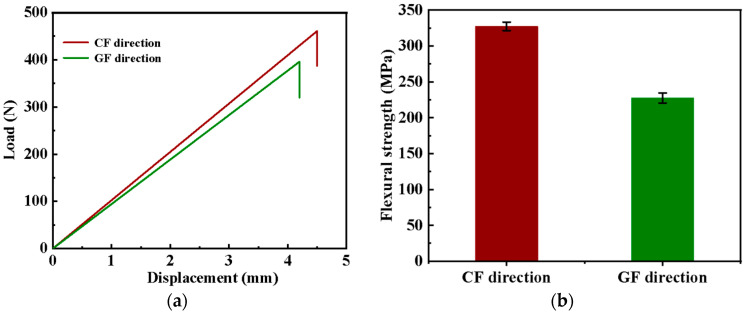
(**a**) The displacement–load curves and (**b**) flexural strength of the GCF/EP along different longitudinal fiber directions.

Figure 1 shows the load–displacement and flexural strength of the GCF/EP in different longitudinal fiber directions. It is evident that the flexural strength of the GCF/EP-CF (327.3 MPa) is much higher than that of the GCF/EP-GF, which is merely 227.5 MPa. The larger flexural strength of the GCF/EP-CF could be related to the superior tensile properties of CFs in comparison with GFs. The flexural strength of the GCF/EP composites in this work is significantly greater than that of GF/EP composites fabricated with the same number of glass fabric layers (170.29 MPa) [31], and it is comparable to the value reported in our previous work on GCF/EP laminates [3].

### 2.2. Electrically Heated Behavior

**Figure 2:**
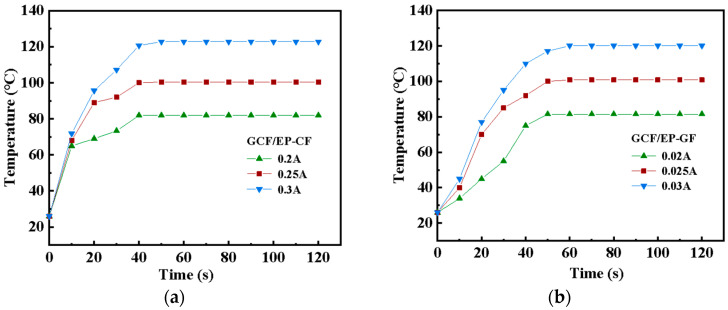
Variation in surface temperature of GCF/EP over time along different current loading directions: (**a**) longitudinal CF direction; (**b**) longitudinal GF direction.

To quantitatively analyze the electrothermal behavior of the GCF/EP specimens, their surface temperature changes were monitored in real time using an infrared thermal imager under various current loads along both the longitudinal CF and GF directions. As depicted in Figure 2, the surface temperature response is swift due to the efficient conversion between electrical and thermal energy under the electrothermal effect of the composite plate. Typically, the temperature rises rapidly in the initial stage before reaching its surface equilibrium temperature, and the rate of temperature response increases with the current magnitude. As illustrated in Figure 2a, when a current is applied along the longitudinal CF direction, the GCF/EP specimen attains the steady-state maximum temperature, also known as the “surface equilibrium temperature”, after around 40 s, which is much shorter than the time required along the longitudinal GF direction (60 s). Furthermore, compared to the longitudinal GF direction of the GCF/EP laminate, where only 0.03 A is needed to achieve a surface equilibrium temperature of 120.1 °C, a stronger current (0.3 A) is required along the longitudinal CF direction to reach a comparable surface equilibrium temperature (122.8 °C). The substantially reduced current magnitude that is needed along the longitudinal GF direction (one tenth of that required along the CF direction) could represent a significant advantage for the design and development of an electrical heater. This suggests that a single structural material can meet various practical demands to reach the same electrical heating temperature, such as fast heating or weak current loading. For instance, the GCF/EP composite plate could be designed and utilized in the structures where its longitudinal CF direction supports the primary load-bearing function, and the longitudinal GF direction is employed for its electrothermal function when loaded with a weak current. The optimal operating current of 0.3 A in the longitudinal CF direction for the GCF/EP composite laminate in this study aligns with that for the CF-reinforced composite [32]. 

Moreover, the time-dependent temperature increase can be empirically expressed as follows [33,34,35]:

(1) $$\frac{T_{t}-T_{0}}{T_{e}-T_{0}}=1-\exp\left(\frac{-t}{\tau_{g}}\right)$$

where T_0_ and T_e_ represent the initial and maximum temperatures, respectively. T_t_ is an arbitrary temperature at time t. The characteristic growth time constant is denoted by τ_g_. Values of τ_g_ were determined by fitting the data from the first region of the temperature versus the time plots in Figure 2, with the results being tabulated in Table 1. Τ_g_ values are lower for GCF/EP-CF compared to those for GCF/EP-GF. This indicates that the temperature responsiveness of the current loading along the longitudinal CF direction of the GCF/EP is better than that along the longitudinal GF direction. With the increasing current magnitude along the same longitudinal fiber direction, the τ_g_ value of the GCF/EP experiences a gradual increase. The average τ_g_ values for the GCF/EP-CF and GCF/EP-GF are 13.47 ± 1.93 and 20.35 ± 2.70, respectively. It has been reported that the overall average τ_g_ of the conductive polypyrrole/poly(vinyl alcohol-co-ethylene) nanofiber composites [36] is 46.7 ± 7.7 s, which is more than three times larger than that of the GCF/EP-CF in this work. 

**Figure 3:**
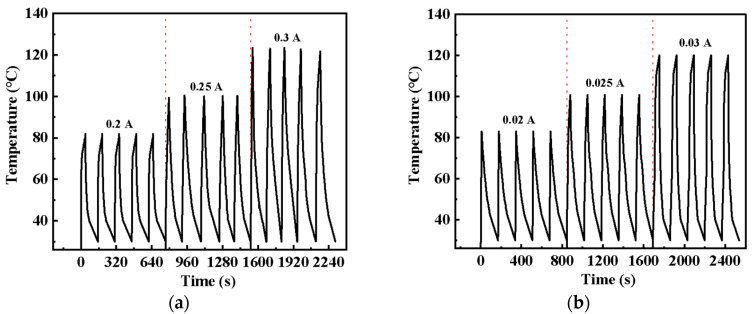
Temperature response of GCF/EP under cyclic current loading with stepwise on/off patterns along different longitudinal fiber directions: (**a**) CF; (**b**) GF.

As an electrical heater, the multi-cyclic electrical heating–cooling behavior of the GCF/EP is also important. Figure 3a,b illustrate the cyclic temperature response of GCF/EP under stepwise current loading along the longitudinal CF and GF directions, respectively. The heating–cooling behavior of the GCF/EP remained constant along both the longitudinal CF direction and the longitudinal GF direction under the same current loading, demonstrating the excellent current rate stability and robust structural stability of the GCF/EP heater in both longitudinal fiber directions. It is evident that the GCF/EP can be used stably at high temperatures (up to 120 °C) and shows great potential as an engineering material in engineering structures for simultaneous deicing applications.

### 2.3. Deicing Performance

The deicing performance of engineering materials in structures is one of their crucial functions to ensure safe operations in icy environments. For example, icing in critical areas such as aircraft wings, tails, and control surfaces can disrupt the airflow and result in a loss of lift and control, posing a significant risk to the aircraft’s serviceability [16]. In addition, the accumulation of ice can increase the overall weight of the aircraft, thereby significantly deteriorating its overall performance and boosting fuel consumption and operational costs. 

To quantify the impact of the GCF/EPs’ electrothermal effect on their deicing performance, a “deicing factor” was proposed to characterize the energy efficiency of the deicing process. This method assumes that the heat generated by Joule heating is completely absorbed by the ice, causing its temperature to rise and the ice to eventually melt. The energy consumed by the composite can be expressed as the generation of heating power and the duration from the start of current loading to the complete melting of the ice. 

According to work reported previously [37,38], the amount of heat that is absorbed by the ice before it melts (Q_1_) can be expressed by Equation (2):

(2) $$Q_{1}=C_{ice}\cdot m_{ice}\cdot \Delta T$$

where C_ice_ is the specific heat capacity of ice (2.1 J/g°C), m_ice_ is the mass of the ice (m_ice_ = 0.4 g), and Δ*T* is the temperature difference from the start of the experiment to the melting point of ice (Δ*T* = 8 °C).

The heat that is absorbed during the ice melting process (Q_2_) is expressed by Equation (3):

(3) $$Q_{2}=m_{ice}\cdot \Delta H_{fus}$$

where m_ice_ is the ice mass, and Δ*H_fus_* is the melting enthalpy for water (335 J/g).

The electrical energy that is consumed during deicing (W) can be expressed by Equation (4):

(4) $$W=I\cdot U\cdot t_{deice}$$

where I is the constant current that is passed through the GCF/EP composite, U is the voltage drop across the GCF/EP, and t_deice_ is the whole current loading time for deicing.

Therefore, the deicing factor (or energy efficiency) can be defined by Equation (5):

(5) $$\eta_{ice}=\frac{Q_{1}+Q_{2}}{W}\times 100\%=\frac{C_{ice}\cdot m_{ice}\cdot \Delta T+m_{ice}\cdot \Delta H_{fus}}{I\cdot U\cdot t_{deice}}\times 100\%$$

**Figure 4:**
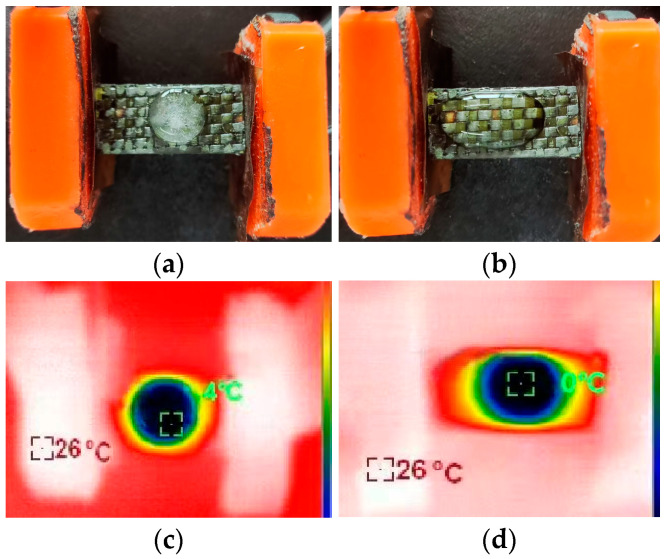
GCF/EP deicing test setup: (**a**) before and (**b**) after deicing process. Thermal imaging of the ice melting process of GCF/EP-CF under 0.3 A current loading at (**c**) 0 s and (**d**) 107 s.

The typical deicing test configuration before current loading and after complete deicing is shown in Figure 4a,b, respectively. The corresponding IR thermal images captured during the test are presented in Figure 4c,d. Under a current of 0.3 A, all the ice on the GCF/EP composite plate rapidly melted to water within 107 s (Figure 4d). The times required for the ice to completely melt into water under various deicing conditions are listed in Table 2. Obviously, when the current is applied along the same longitudinal fiber direction, the deicing time decreases with the increasing magnitude of the current. For instance, with a 0.2 A current being applied along the longitudinal CF direction, the resulting GCF/EP-CF took 132 s to completely melt the ice. As expected, the deicing process of the GCF/EP-CF can easily be shortened by applying stronger currents. At 0.3 A, the corresponding deicing time was reduced to 107 s (Table 2). This correlates with the surface equilibrium temperature that is achieved under different current loadings along the longitudinal CF direction (Figure 2a), as the ice on the high-temperature electric heater melts quickly. However, it is noteworthy that once the current loading direction was switched to the longitudinal GF direction, the resulting GCF/EP-GF took 153 s to melt ice at a current load of 0.03 A, despite achieving a similar surface equilibrium temperature to that along the longitudinal CF direction at 0.3 A (Figure 2b). Although the GCF/EP takes less time to melt ice with stronger currents along the longitudinal CF direction, a much weaker current could also complete the deicing process by simply switching the current loading to the longitudinal GF direction. This could be advantageous in scenarios that favor the usage of a weaker current. It is evident that energy is required to melt the same amount of ice. Generally, the stronger the current that is applied to the electrical heater is, the more thermal energy is consumed by the deicing process. However, it should be noted that at higher current loads, the output Joule energy may not be as high as expected based on Joule’s law (Q = W = UIt) when the constant current is applied in different directions of the GCF/EP composite laminate. The deicing factor of the GCF/EP plate increases with the loading current in both longitudinal CF and GF directions, indicating the lower energy efficiency in the deicing process at smaller current magnitudes. This may be due to the fact that more heat is lost or dissipated to the surroundings at lower current loads, as it always takes a longer time to complete the deicing process. All the deicing factors of the GCF/EP-GF are lower than those of the GCF/EP-CF, despite the similar surface equilibrium temperatures in the two longitudinal fiber directions. The deicing factor of the GCF/EP-CF at 0.3 A is 82.71%, which is the largest among all electrical heating conditions. This is slightly lower than the deicing efficiency reported for carbon fiber/poly ether ether ketone composites, where the deicing process was achieved by the ice layer falling off from the surface of the specimen [39], as opposed to the ice completely melting in this work. Wang et al. [40] reported that by introducing two CNT webs to the CFRP surface and its adjacent interface, the time required for the complete melting of the ice into water could be reduced from 33 min to 10 min. In contrast, the deicing time in this work could conveniently be adjusted within 107s and 203 s by tuning the current magnitude and its loading direction. These results confirm the anisotropic deicing performance of the GCF/EP composite laminate.

**Figure 5:**
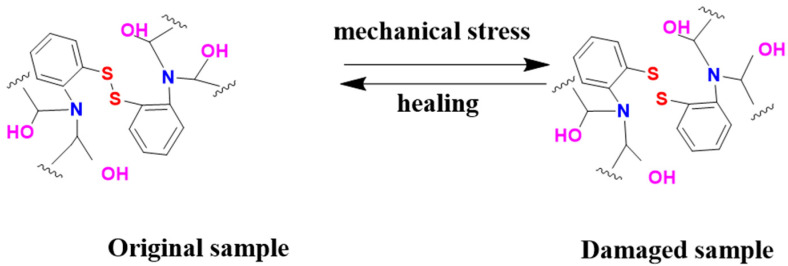
The self-healing mechanism of the epoxy resin.

**Figure 6:**
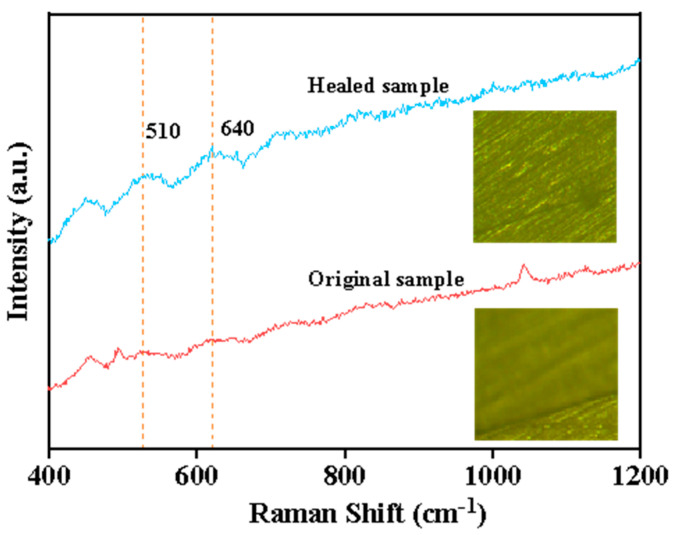
Raman spectrum of the fractured epoxy sample before and after the healing process, with insets showing their optical images.

The disulfide bond in the curing agent 2-AFD that was utilized in this study afforded self-healing capability to the epoxy composites, and their self-healing mechanism is illustrated in Figure 5. To elucidate the role of dynamic disulfide bonds in the self-healing mechanism of epoxy materials, Raman spectroscopy was employed to analyze the chemical changes at the crack site before and after the healing process. As shown in Figure 6, two peaks related to the S-S bonds in the epoxy resin could be observed, where the peak centered at 510 cm^−1^ originated from the disulfide bonds and the one at 640 cm^−1^ was from the C-S bonds [23,41]. Moreover, in comparison with the thick crack in the original fractured epoxy sample (inset optical image in Figure 6), it diminished in the healed sample after subjection to the self-healing process at 120 °C for 2 h, and the characteristic peak of the S-S bonds became more pronounced, confirming the healing of the epoxy resin sample. 

**Figure 7:**
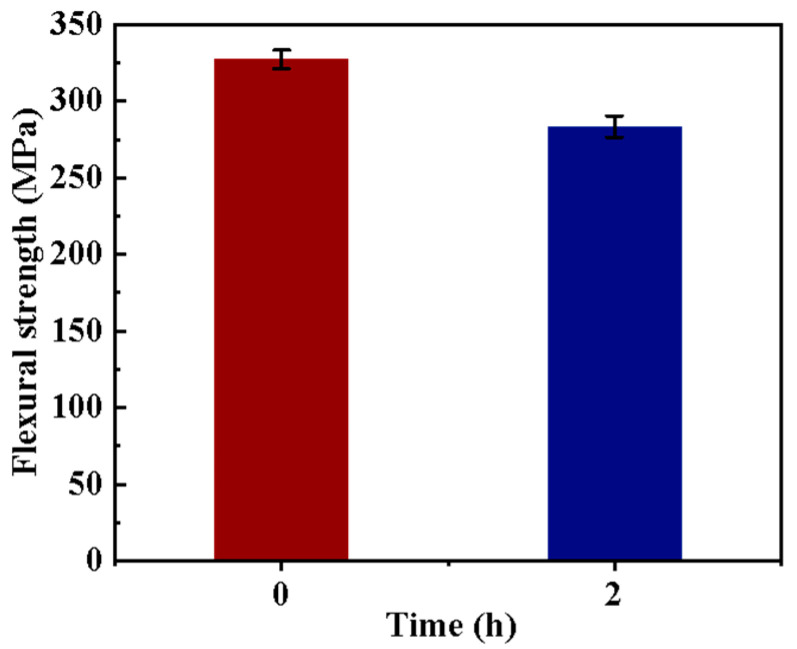
Flexural strength of the original and healed GCF/EP-CF composite.

The self-healing effect of the GCF/EP on both the bending properties and the deicing performance were also investigated. Considering the superior mechanical property and deicing performance of the hybrid fiber-reinforced composite laminate along the longitudinal CF direction, the study of the self-healing properties of the GCF/EP was only conducted in this direction. As shown in Figure 7, the flexural strength of the healed composites is about 283.5 MPa, which signifies that 86.6% of the original strength has been recovered. Regarding the deicing performance, as shown in Table 3, both the electric resistance and the deicing performance of the healed samples change little compared to the original intact ones, suggesting the potential for practical application of the self-healing GCF/EP composite laminate.

**Figure 8:**
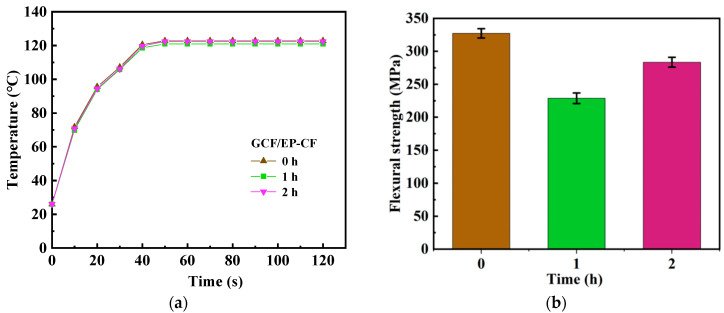
(**a**) The electrothermal performance and (**b**) the mechanical properties of GCF/EP-CF after healing for different durations.

Furthermore, the electrothermal performance and mechanical properties of the hybrid fiber-reinforced epoxy composites, which had been healed for various durations, were investigated. As shown in Figure 8a, after healing for 1 h, the sample displayed negligible change in its electrothermal performance compared to the one that had been healed for 2 h. However, its restoration of flexural strength was notably poorer, as shown in Figure 8b.

### 2.4. Micro-Structural Analysis

**Figure 9:**
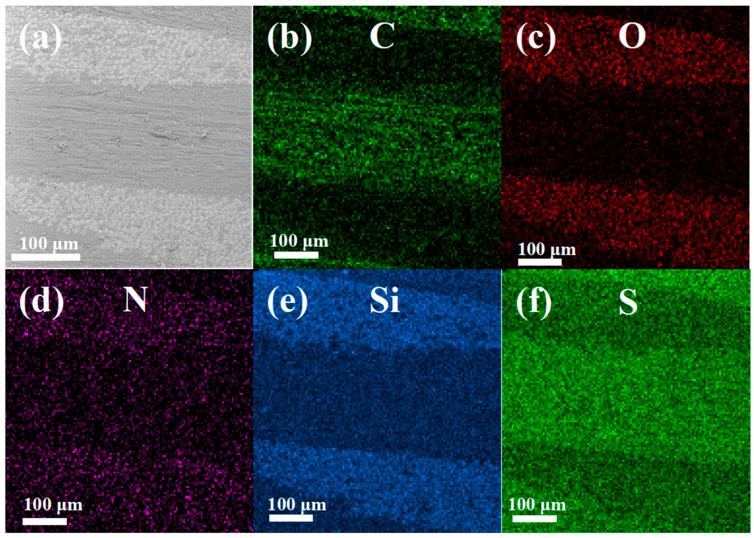
EDS mapping analysis of GCF/EP composite plates: (**a**) SEM image and elemental maps of (**b**) C, (**c**) O, (**d**) N, (**e**) Si, and (**f**) S.

To further explore the internal structure of the GCF/EP-CF, SEM-EDS mapping analysis was performed on its cross-section to ascertain the elemental distribution and identify the CF and GF regions of the samples (Figure 9). The orientation of the GF could be verified by Si mapping (Figure 9e) in conjunction with Figure 9a and was shown to be perpendicular to the cross-section and manifest as circular dots (the cross-section of GFs) in the images. Meanwhile, the CFs are aligned horizontally from left to right in the images (Figure 9a,b). However, due to the embedding of the CFs in the epoxy matrix and the C element being indistinguishable from the CFs and epoxy matrix, it is challenging to delineate the epoxy-rich area in the images. Interestingly, despite the fact that the epoxy matrix was cured with the hardener 2-AFD, which should provide a uniform distribution of N throughout the resin area, a higher concentration of N appears in the GF regions (Figure 9d,e). This suggests the presence of a sizing agent containing N groups on the GFs.

**Figure 10:**
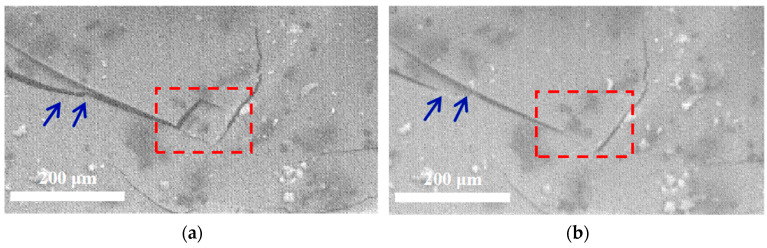
SEM images of GCF/EP-CF subjected to a bending test (**a**) before and (**b**) after healing.

After conducting the three-point bending test, the surface of the GCF/EP-CF was also observed and analyzed both before and after the self-healing treatment (120 °C for 2 h). Figure 10a,b illustrate the variation in cracks on the top surface of the specimen before and after healing, respectively. It is evident that the micro-cracks (highlighted by a red box and blue arrows) on the surface of the specimen in Figure 10a have been healed, although some larger cracks persist (as seen in Figure 10b). This indicates that achieving complete healing of the composite is challenging.

## 3. Materials and Methods

### 3.1. Preparation of Glass Fiber/Carbon Fiber Epoxy Resin Composites

**Figure 11:**
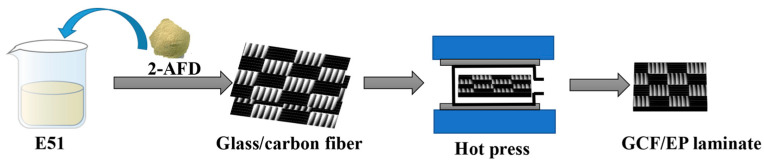
Schematic of the fabrication of the GCF/EP composite laminate.

**Figure 12:**
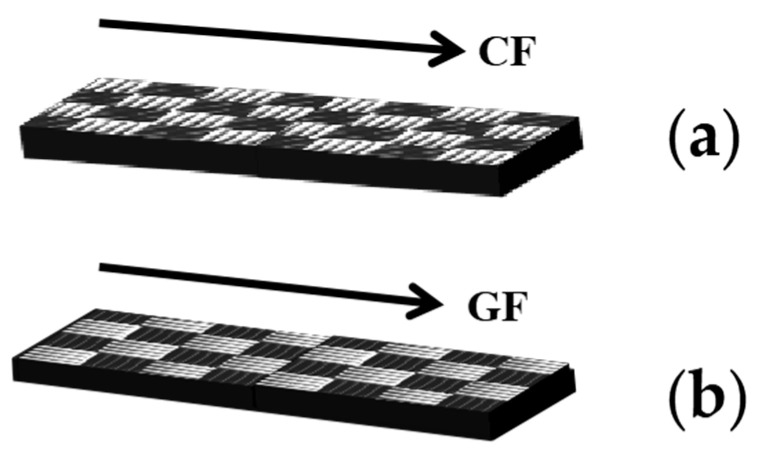
Schematic of GCF/EP composite samples along different longitudinal fiber directions: (**a**) GCF/EP-CF; (**b**) GCF/EP-GF.

Hybrid composite laminates with dimensions of 150 mm × 140 mm were prepared using plain woven glass/carbon fabrics (Huarike New Materials Co., Ltd., Changzhou, China). The area density of the hybrid fabric is 251.9 g/m², and the volume ratio of CF:GF is about 1:1.7. Epoxy resin E51 from Zhonggao Chemical Co. Ltd. (Guangzhou, China) was used as the matrix, and the curing agent was 2,2′-diaminodiphenyl disulfide (2-AFD, Shanghai Bide Pharmaceutical Technology Co. Ltd., Shanghai, China). The entire manufacturing process of the GCF/EP composite is shown in Figure 11. E51 and 2-AFD were mixed in a mass ratio of 2:1. The composites were fabricated in vacuum bags by the hand layup method. Hybrid composite laminates were prepared with 8 fabric layers, which were infiltrated with the epoxy mixture and subsequently cured in a vacuum bag at 120 °C for 2.5 h. During the curing process, a pressure of 10 MPa was provided by a hot press. The hybrid fiber volume fraction in the GCF/EP composites was 47.9%. GCF/EP specimens with dimensions of 15.0 mm (width) × 2.7 mm (thickness) × 60.0 mm (length) were prepared for mechanical testing. To study the anisotropic properties of the composite laminate, specimens with the lengthwise direction of the GCF/EP rectangular plate, aligned with the longitudinal CF direction, were labeled with “GCF/EP-CF” (Figure 12a), whereas those that were aligned with the longitudinal GF direction were labeled with “GCF/EP-GF” (Figure 12b).

### 3.2. Test Instrument and Method

Static three-point bending tests were performed on a universal testing machine (model UTM5105, Shenzhen Sansi Zongheng Technology Co., Ltd., Shenzhen, China) according to ASTM D7264. For all bending tests, the cross-head speed was set to 2 mm/min. The flexural strength (σ) was calculated using Equation (6):

(6) $$\sigma =\frac{3\mathrm{PL}}{2{\mathrm{bh}}^{2}}$$

where σ is the flexural strength, P is the load applied at the center of the span, L is the support span (48 mm), and b and h are the width (15 mm) and thickness (2.7 mm) of the specimen, respectively.

For the electrothermal tests, both cross-sections of the GCF/EP panels were polished and connected to a copper sheet, which also served as the electrode. To minimize the contact resistance, a high-temperature conductive adhesive was applied between the Cu sheet and the GCF/EP specimens. During the tests, the GCF/EP composites were loaded with currents of 0.2 A, 0.25 A, and 0.3 A along the longitudinal CF direction (the corresponding stable resistances were measured to be 17.67 Ω, 23.72 Ω, and 34.45 Ω), while currents of 0.02 A, 0.025 A, and 0.03 A were applied along the longitudinal GF direction (the measured stable resistances were 2740 Ω, 1772 Ω, and 1479 Ω). The temperature was monitored with an infrared thermal imager (Xinst HT-19, Dongguan, China). For the deicing test, the same amount of water (0.4 g) was frozen in a refrigerator. After loading different currents, the time required for the complete melting of the ice was recorded and compared. Zeiss ultra plus SEM (Oberkochen, Germany) was used to observe the cross-section surfaces of the composites, which were sputter-coated with a thin Au layer prior to observation. A micro-Raman spectrometer (LabRAM HR Evolution, HORIBA Scientific Instruments Division, Palaiseau, France) was used to analyze the physical chemical structure of the epoxy samples. For the healing test of the GCF/EP composite, specimens that had undergone bending tests were heated in an oven at 120 °C for 2 h. All mechanical property test results were determined using at least three independent specimens to ensure statistical reliability.

## 4. Conclusions

In this work, the anisotropic bending properties, electrothermal properties, and deicing performance of a self-healing GCF/EP composite are investigated. The findings are as follows:

1. The flexural strength of the GCF/EP along the longitudinal CF direction was 43.87% higher than that in the longitudinal GF direction. Additionally, the self-repair rate of the GCF/EP-CF could reach 86.6% after being healed for 2 h at 120 °C.

2. Applying different currents in various directions of the GCF/EP laminate composites showed excellent cyclic heating-cooling stability. The laminate with current loading along the longitudinal CF direction was capable of reaching an equilibrium temperature within 40 s, which was faster than that of its counterpart along the longitudinal GF direction. Moreover, the average τ_g_ value of the GCF/EP along the longitudinal CF direction was much lower than that along the longitudinal GF direction, indicating their rapid electrical heating and deicing behavior.

3. The deicing characteristics of the GCF/EP along both the longitudinal CF and GF directions increased with the current magnitude. A weaker current for the deicing process could be achieved by changing the current loading along the longitudinal GF direction. Meanwhile, the shortest deicing time and largest deicing factor of the GCF/EP could be achieved by applying the current along the longitudinal CF direction.

4. After undergoing self-healing treatment, the flexural strength and deicing performance of the GCF/EP could be restored by 86.6% and 81.37%, respectively.

## Figures and Tables

**Table 1:** The electrothermal performance of the GCF/EP under various current loadings along different fiber orientations.

Sample	Applied Current (A)	Equilibrium Temperature (°C)	τ_g_ (s)
GCF/EP-CF	0.2	81.9	12.19 ± 3.83
	0.25	100.4	12.20 ± 1.55
	0.3	122.8	16.03 ± 0.40
GCF/EP-GF	0.02	81.5	18.65 ± 2.31
	0.025	100.8	20.62 ± 1.93
	0.03	120.1	21.77 ± 3.87

**Table 2:** Deicing behavior of GCF/EP composite laminate under various current loadings along longitudinal CF and GF directions.

Sample	I (A)	U (v)	t_deice_ (s)	η_deice_ (%)
GCF/EP-CF	0.2	6.89	132	77.36
	0.25	5.93	120	79.10
	0.3	5.30	107	82.71
GCF/EP-GF	0.02	54.80	203	63.25
	0.025	44.29	190	66.89
	0.03	44.37	153	69.55

**Table 3:** Electrical, mechanical, and deicing properties of the original and healed GCF/EP-CF composite sample.

Sample	Resistance (Ω)	Flexural Strength (MPa)	η_deice_ (%)
GCF/EP-CF	21.4	327.3	82.71
GCF/EP-CF1	23.2	283.5	81.37

## Data Availability

The original contributions presented in the study are included in the article; further inquiries can be directed to the corresponding authors.
